# Effect of semantic distance on learning structured query language: An empirical study

**DOI:** 10.3389/fpsyg.2022.996363

**Published:** 2022-11-09

**Authors:** Shin-Shing Shin

**Affiliations:** Department of Information Science and Management Systems, National Taitung University, Taitung, Taiwan

**Keywords:** semantic network theory, semantic distance, structured query language, learning, semantic transformation

## Abstract

Students of database courses usually encounter difficulties in learning structured query language (SQL). Numerous studies have been conducted to improve how students learn SQL. However, learning SQL remains difficult. This study analyzed the difficulties in learning SQL from the viewpoint of semantic distance by using semantic network theory. An experiment involving a database course was performed to assess the influence of semantic distance on learners’ understanding of SQL. The participants were requested to perform a query-writing task at the end of the course to investigate their understanding of SQL. The data analysis results indicated that the participants developed a better understanding of the formulation-to-planning transformation than the planning-to-coding transformation. This implies that the semantic distance of the planning-to-coding transformation is greater than that of the formulation-to-planning transformation, and the semantic distance of the planning-to-coding transformation is attributable to the semantic transformation from natural language to SQL, which are two essentially different languages and belong to different knowledge categories. Accordingly, this study concludes that SQL learning difficulties can mainly be ascribed to the planning-to-coding transformation because the large semantic distance. The findings suggest that SQL instructions should emphasize the semantic mapping of the planning-to-coding transformation by incorporating materials related to the transformation and should shorten the semantic distance involved in learning SQL. These two principles can be used to evaluate the effectiveness of SQL teaching methods in assisting SQL learning, and motivate researchers to develop more effective teaching methods from the viewpoint of semantic distance.

## Introduction

Structured query language (SQL) is an essential part of database courses. However, learning SQL is challenging (Taipalus et al., 2018; Taipalus, 2020b; Taipalus and Seppänen, 2020). Studies have demonstrated that the following difficulties are prominent in learning SQL. First, the declarative syntax of SQL renders the execution steps invisible (Sadiq et al., 2004). Learners have to visualize the execution of SQL statements in their mind, which may be cognitively burdensome, thereby lowering learning outcomes. Studies have revealed that visualizing the execution process is difficult for learners (Lavbič et al., 2017). The second is complex semantics, where studies have revealed that SQL syntax’s simple appearance conceals its complex semantics, especially for the aggregate function, grouping, restricting grouping, correlated subquery, multitable join, self-join, and nested-type query (Hardt and Gutzmer, 2017). Users are overwhelmed by the complexity of using these concepts to solve data queries.

Pedagogical research on SQL has led to the development of numerous SQL tutoring systems and pedagogical methods. Some of them focus on illustrating the process of how SQL statements are executed to reduce the cognitive load of SQL learners; these methods include eSQL (Kearns et al., 1997), SQL Advanced VI-sualization (SAVI) (Cembalo et al., 2011), Database Query Analyzer (DBQA) (Hardt and Gutzmer, 2017), ADVICE (Cvetanovic et al., 2011), and concept-map-based SQL instructions (Shin, 2020). Some of these methods provide learners with personalized instruction [e.g., SQLator (Sadiq et al., 2004), SQLT-Web (Mitrovic, 2003), and Acharya (Bhagat et al., 2002)] and intelligent feedback [e.g., LEARN-SQL (Abelló et al., 2008), SQLify (Dekeyser et al., 2007), SQL-LTM (Dollinger, 2010), and SQL-Trainer (Laine, 2001)], and other methods automatically generate the SQL statements of data queries to help learners understand SQL, such as SQL in Steps (SiS) (Garner and Mariani, 2015) and SQL Developer (Narayanan, 2016).

The above tutoring systems and pedagogical methods have promoted research on SQL learning. However, studies have indicated that SQL is still hard to understand (Taipalus, 2020a; Taipalus and Seppänen, 2020). To enhance the development of SQL pedagogies, the causes underlying these challenges must be clarified. Thus, the present study adopted a semantic network theory perspective (Quillian, 1967)—where the learning of SQL is considered to primarily involve a semantic transformation from data requests to SQL statements—something few studies have done. This study also contributes to the establishment of a theoretical foundation for evaluating the effectiveness of SQL pedagogical methods and tutoring systems. Given these considerations, the topics that must be addressed are how learners transform the semantics of data requests into SQL statements and how the semantic distance involved in semantic transformation affects a learner’s understanding of SQL. This study conducted an empirical experiment in a database course. The participants were requested to perform a query-writing task at the end of the course to investigate their semantic transformation processes and examine the effects of the semantic distance on SQL learning.

## Literature review and hypothesis development

This section first reviews SQL tutoring systems and pedagogical methods, then introduces the cognitive model of SQL learners, and finally develop the hypothesis of this study accordingly.

### Tutoring systems

A review of the literature revealed that the characteristics of tutoring systems include feedback on query semantics, distance learning, correctness checking, personalized instructions, execution process, gamification, database schema, and SQL statement generator. In feedback on query semantics, the semantic and syntactic errors of learners’ SQL statements are explained. In distance learning, a web-based learning environment is provided. In correctness checking, the correctness of learners’ SQL statements is determined. In personalized instruction, learning records are collected and personalized instructions are then assigned to the learners according to their learning status. The execution process involves graphically presenting the process of executing SQL queries. In gamification, game elements, such as points, badges, and leaderboards, are used to learn SQL. Database schema involves displaying the database schema for answering SQL-writing questions. In SQL statement generator, SQL statements of data requests are automatically generated and presented to the learners. Table 1 summarizes the characteristics of these tutoring systems. Some of these systems are introduced as follows.

**TABLE 1:** An overview of tutoring systems.

System	Feedback on query semantics	Correctness checking	Distance learning	Personalized instruction	Execution process	Gamification	Database schema	SQL statement generator
SQLT-Web	Yes	Yes	Yes	Yes	No	Yes	Yes	No
eSQL	No	Yes	No	No	Yes	No	Yes	No
SAVI	No	Yes	Yes	No	Yes	No	No	No
DBQA	Yes	Yes	Yes	No	Yes	No	Yes	No
SiS	No	Yes	Yes	No	No	No	Yes	Yes
SQL Developer	No	Yes	Yes	No	No	No	Yes	Yes
ITSB	No	Yes	No	Yes	No	No	No	No
SQL Tester	No	Yes	Yes	No	No	No	Yes	No
AsseSQL	No	Yes	Yes	Yes	No	No	Yes	No
eLGuide	No	No	Yes	Yes	No	No	No	No
YASQLT	Yes	Yes	Yes	No	No	No	Yes	No
SQL visualizer	NA	NA	No	No	No	No	Yes	Yes
QUERY	No	Yes	Yes	No	No	Yes	Yes	No
EDB	Yes	Yes	Yes	No	No	No	No	No
Fujita et al., 2019	No	Yes	Yes	Yes	No	No	No	No
SQLator	No	Yes	Yes	Yes	No	No	Yes	No
SQLify	Yes	Yes	Yes	No	Yes	No	Yes	No
LEARN-SQL	Yes	Yes	Yes	No	No	No	Yes	No
ADVICE	Yes	Yes	Yes	Yes	Yes	No	Yes	No
Acharya	Yes	Yes	Yes	Yes	No	No	Yes	No
SQL-Trainer	Yes	Yes	Yes	Yes	No	No	No	No
SQL-LTM	Yes	Yes	Yes	No	No	No	No	No

eSQL (Kearns et al., 1997) is one of the earliest animated SQL learning tools wherein the execution process of an SQL statement is broken down into multiple steps and displayed sequentially in animated form. The clause executed in each step is detailed. The tables, columns, rows, and cells involved in each step are marked to emphasize the part of the data that the clause is processing. During the animation presentation process, learners can proceed either sequentially or directly to the final result. Similar to eSQL, in SAVI (Cembalo et al., 2011), the execution steps of an SQL statement are illustrated one at a time. The difference between the two tools is that SAVI focuses on explaining the meaning of SQL operators and how the data are operated by the operators, whereas eSQL emphasizes presenting the evolutionary sequence of datasets. In SAVI, the functionality of eSQL is expanded through reversible animation, which allows learners to backtrack. Furthermore, SAVI is a web-based learning environment. DBQA (Hardt and Gutzmer, 2017) provides animations similar to those in SAVI and eSQL. Learners can move forward or backward in the evolution of the datasets. Moreover, DBQA supports subqueries, which are usually difficult for learners to understand. Because the default error messages provided by database systems are difficult to comprehend, DBQA converts the original messages of database systems into user-friendly messages.

SQLT-Web (Mitrovic, 2003) was the first constraint-based system to support students in learning SQL. It tailors instructional actions for individual students. Students learn SQL by answering SQL-writing questions, the difficulty of which depends on their learning status. SQL-Tutor builds a student model for each student to record the student’s learning status, including their SQL knowledge level, learning abilities, and general characteristics. When students input a solution to a question, SQL-Tutor analyzes the solution through hundreds of constraints to provide students with semantic and syntactic error messages and updates their state of learning. One of the main advantages of SQL-Tutor is that, in addition to providing feedback on the syntax, the system also provides meaningful feedback on the semantic correctness of the students’ solutions. Mitrovic (2003) published a series of SQL learning–related research, such as the effect of gamification on SQL learning (Tahir et al., 2020).

SQL in Steps (Garner and Mariani, 2015) aids the learning of SQL by automatically generating the SQL statement of data requests. SiS comprises a graphical query designer and an SQL translator. In SiS, learners are guided to build SQL queries in a step-by-step manner on the graphical query designer. Each change by learners in the query designer triggers the SQL translator to generate the corresponding SQL statement and refresh the query result.

e-Learning Guide (eLGuide) (Zafar and Albidewi, 2015) is a web-based SQL learning system that mainly utilizes fuzzy theory to provide students with personalized instructions. eLGuide consists of an information retrieval module, a student module, and an advice generation module. The information retrieval module retrieves the most relevant material for a particular concept that a student is currently learning. The student module constructs a model of the knowledge, preferences, and goals for each student. As students interact with the course material, their respective student models are continuously updated. The advice generation module personalizes the learning path for each student according to their student model.

Yet Another SQL Tutor (YASQLT) (Bider and Rogers, 2016) is an automated assessment tool to help novice learners enrolled in an introductory database course. YASQLT focuses on providing learners with feedback on common semantic errors. YASQLT evaluates learners’ SQL queries by comparing their answers with the dataset generated by the instructor’s SQL query. If the result is incorrect, YASQLT gradually adds checks to identify possible common errors and provide feedback. YASQLT provides several questions on different themes, such as simple select, join, union, group by, and group by. The questions are randomly assigned to learners.

QUERY (Barla et al., 2016) is a web-based application that provides an interactive SQL learning environment through automatic evaluation of SQL queries. QUERY is implemented in a service-oriented architecture in order to foster the reusability and modularity of the tool. QUERY includes predefined exercises. Instructors can also create exercises and schema. The evaluation of a learner’s attempts at a solution is performed by comparing the resultant datasets of the learner with that of the instructor. When learners find that the assignments are unclear or that errors are present in the solutions, they can send feedback to their instructors. Instructors can view the overall activity and performance of a class and identify exercises that are deemed difficult by the learners. Learners can also see how they perform relative to their classmates.

Obaido et al. (2018) developed an interactive visualization tool, SQL visualizer, to aid the learning of SQL. This tool provides database schemas, a query box, and an SQL query generator. To reduce the cognitive load of SQL learners, the tables and attributes of database schemas and SQL operators are represented as icons. The query box is used to specify the tables and attributes required in an SQL query. The SQL query generator then generates an SQL statement based on the icons in the query box. Learners drag and drop icons into the query box and the corresponding SQL statement is then automatically generated by the SQL query generator. When icons in the query box cannot generate an SQL statement, a textual suggestion is provided to explain the possible reasons.

Intelligent Tutoring System Builder (ITSB) (El Agha et al., 2018) is a SQL tutor designed for novice learners. ITSB comprises a student module, a domain knowledge module, and a pedagogical module. The student module collects the learning information of each student, including lessons, the number of exercises, knowledge level in each learning objective, scores, and date. The domain knowledge module stores 16 lessons, which contain many themes of SQL. The pedagogical module decides when and what learning information should be provided to students based on the student module. ITSB provides exercises on different themes, and the students must answer these exercises correctly to move to the next theme.

SQL Tester (Kleerekoper and Schofield, 2018) is similar to AsseSQL (Prior, 2014), with some differences in terms of the number of questions, question categories, randomization of question order, and presentation of database schema. SQL Tester provides learners with an SQL query-writing test. Learners must answer 10 questions within 50 min. The questions are randomly selected from the question repository, which contains nine question categories and each category contains four to eight questions. During a test, learners can answer questions in any order and are allowed multiple attempts before submitting. After each attempt, the tool presents the rows returned or the corresponding error message.

Fujita et al. (2019) developed a web-based learning system to support the SQL exercises. In this tool, a log function records the learning history of each learner. Learners can view the 20 most recently executed SQL statements and their errors based on the learning history. Instructors can view a learner’s learning activity, including the number of executed SQL statements, usage time, and login information to analyze the learner’s understanding of SQL, thus enabling the instructor to provide appropriate instruction to each learner.

EDB (Faeskorn-Woyke et al., 2020) is a web-based e-learning platform for improving the first step of SQL learning. EDB constructs a decision tree to classify SQL errors and provides personalized feedback based on this to help learners avoid these errors. EDB collected 7,353 wrong solutions of learners as a training set for building the decision tree, which has 106 leaf nodes. Each leaf node represents a similar type of error, which is used to generate an error message and feedback to help learners improve.

### Pedagogical methods

Some pedagogical methods have been developed to help alleviate SQL learning difficulties. Qian (2018) developed a systematic approach for constructing SQL queries based on the divide-and-conquer paradigm. The main idea of this approach comes from problem-solving skills in programming. When performing an SQL query for a data request, this approach decomposes the data request into subrequests that can be easily performed using SQL. The SQL statements of these subrequests are then combined to accomplish the original SQL query. The divide-and-conquer SQL learning approach is achieved by using keyword checklists, patterns, and templates. Learners recognize patterns in data requests according to keywords and then map the patterns into corresponding SQL syntax based on the templates. With this learning approach, learners can construct an SQL query for data requests in a step-by-step manner.

Concept-map-based SQL instruction (Shin, 2020) focuses on reducing the cognitive load of SQL learners by representing the execution process of SQL statements as concept maps. The learners are provided with instructions to guide them to implement SQL queries by using concept maps, which display how the resultant dataset of a data request evolves from the initial datasets and how they are mapped to the SQL statement. After constructing the concept map, the instructors guide the learners to study the concept map prepared by the instructor for the SQL query to assess their understandings.

The aforementioned tutoring systems and pedagogical methods have facilitated SQL learning research. However, learning SQL can still be challenging (Taipalus, 2020b; Taipalus and Seppänen, 2020) because the aforementioned teaching methods have some limitations. For example, animated SQL tutoring systems, (e.g., eSQL, SAVI, and DBQA) can display the intermediate datasets of SQL queries to help learners understand SQL. However, SAVI and eSQL do not support subqueries. Furthermore, because of the limitations of graphical interfaces, they cannot present the execution process of complex SQL queries. Nevertheless, learners need these tutoring systems the most when they are learning complex SQL queries (Ahadi et al., 2015).

For SQL query generators, tools such as SiS and SQL visualizer can automatically generate SQL statements for data requests to help learners learn SQL. However, graphical query builders cannot make highly complex data queries (Garner and Mariani, 2015). For instance, SQL visualizer only supports simple select queries and not advanced SQL syntax (e.g., join, order by, group by, and aggregate functions). SiS has limited support for subqueries, which can only be used in the “from” clause of SQL statements. Furthermore, learners may be accustomed to graphical interfaces and may find it difficult to revert to using textual statements (Renaud and Van Biljon, 2004).

Other examples include SQL Tester, which provides learners with an SQL-query-writing test. However, this tool does not support advanced SQL questions, such as correlated subqueries and self-joins. YASQLT can evaluate learners’ SQL queries and provide feedback on semantic errors; however, it only supports *select* and *create view* statements. All these tools have some limitations. To overcome these limitations, a broader perspective (e.g., semantic distance) is required to clarify the reasons that underlie difficulties in SQL learning to, in turn, improve learning outcomes.

### Cognitive model of structured query language learners

This study combined the findings of Ogden (1985) and Borthick et al. (2001) to explain the cognitive processes and semantic transformation of SQL learners. Borthick et al. (2001) indicated that formulating a data query for accessing databases requires knowledge of three domains, namely data request, data model, and query language. First, learners identify the required constructs from a data request statement. Second, they examine the data model to identify the elements required for obtaining the required constructs. These two processes constitute a mental model for solving the data request. Third, learners translate the mental model into a query language syntax to fulfill the data request.

**FIGURE 1:**
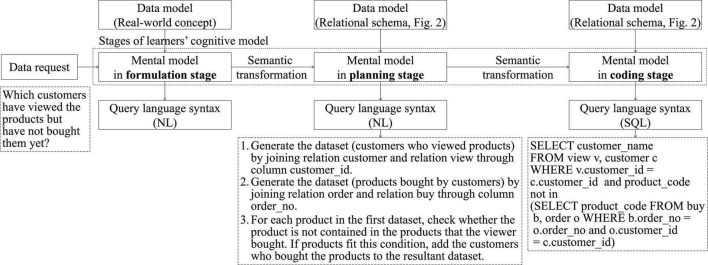
Structured query language (SQL) learners’ cognitive model.

Ogden (1985) developed a three-stage cognitive model of the data query process, which encompassed the formulation, planning, and coding stages. This study combined the model with the findings of Borthick et al. (2001) to describe the cognitive models of SQL learners (Figure 1) and identify the data model and query language required in each stage, and then analyze the semantic transformation between the stages. In the formulation stage, learners make decision on what data they need from a real-world perspective. For example, consider the process of learning the SQL query in Figure 1. First, learners identify required constructs from the data request. Four constructs, namely customer, view, product, and buy, are identified. Second, learners examine their real-world concepts to identify the data required for obtaining these constructs. These two processes result in the development of a mental model for solving the data request. Third, learners translate the mental model into a natural language (NL) syntax to describe the solution. In this stage, learners typically visualize the solution in their minds instead of describing their solution using NL.

**FIGURE 2:**
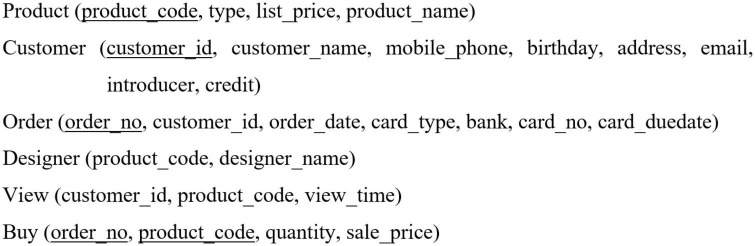
Relational schema of the data request in Figure 1.

In the planning stage, learners transform the output of the formulation stage into a query logic from the perspective of logical data modeling, namely relational models. This process involves a semantic transformation from the knowledge of real-world concepts to the knowledge of relational models. In this example, learners switch from the constructs identified in the formulation stage to the relational schema (Figure 2), including six relations: namely product, customer, order, designer, view, and buy. Based on these six relations and the data request statement, they generate a mental model for solving the data request. Finally, they can translate their mental model into a NL syntax to describe their query logic. A query logic example is displayed in Figure 1. Similar to the formulation stage, this stage has learners typically visualizing their solutions in their minds instead of describing them by using NL.

In the coding stage, learners transform their query logics developed in the planning stage into an SQL statement from the perspective of relational models. This process involves a semantic transformation from NL to SQL. An SQL statement example is presented in the coding stage in Figure 1. For example, the first step of the query logic (generate the dataset “customers who viewed products” by joining relation customer and relation view through column customer_id) is transformed into the SQL statement (SELECT customer_name FROM view v, customer c WHERE v.customer_id = c.customer_id).

Accordingly, when learning SQL queries, the cognitive process of learners involves two semantic transformations, namely formulation-to-planning and planning-to-coding transformations. Analyzing the difference between the two semantic transformations may help determine where, how, and why the semantic distance affects SQL learning. This may also help in laying a theoretical foundation for explaining SQL learning difficulties and analyzing the effectiveness of SQL instruction, thereby promoting the further development of SQL instruction.

### Semantic network theory and semantic distance

Semantic network theory represents semantic memory as a mass of nodes interconnected by links (Quillian, 1967). The concepts learned by humans are represented by nodes. Links represent semantic relationship between the concepts. The semantic distance represents the semantic relationship between the concepts (Gray and Rumpe, 2019). Concepts that belong to similar categories have common properties in the semantic structure. The links between these concepts are thus short and semantically related (Gonzalez, 2019). The longer the semantic distance between two nodes is, the lower the accuracy of recalling the semantic relationship between them is (Ratcliff, 1978). Studies have indicated that understanding is positively correlated with the accuracy of recall (Ashcraft, 2002; Ortony et al., 2022). Therefore, understanding the semantic relationship between concepts belonging to similar knowledge categories is easy. In the context of SQL learning, the data models and languages used in various stages of SQL learners’ cognitive model differs. Thus, the understanding of SQL is affected by the semantic distance between the data models (real-world concept and relational model) and the semantic distance between the languages (NL and SQL).

Regarding languages, the planning-to-coding transformation involves a semantic transformation from NL to SQL, whereas formulation-to-planning transformation does not. This study used NL as a procedural language to describe a query logic step by step, while SQL is a pure declarative language. Procedural languages and declarative languages belong to dissimilar categories. Therefore, the semantic distance of the planning-to-coding transformation is greater than that of the formulation-to-planning transformation.

Regarding data models, the formulation-to-planning transformation involves a semantic transformation from real-world concepts [ex. entity-relationship (ER) model] to relational models, whereas the planning-to-coding transformation does not. An ER diagram can be transformed into a relational schema by using mapping rules (Elmasri and Navathe, 2016). Thus, the semantic mapping between them is specific and precise. When learning the semantic mapping, learners can engage in precise elaborations. Precise elaborations promote the cognitive process of encoding learning materials into the semantic network through the production of effective and stable links (Chi, 2018). Therefore, the semantic distance between real-world concepts and relational models is small.

Considering that the semantic distance between real-world concepts and relational models is small and the semantic distance between NL and SQL is large, this study proposed that the semantic distance involved in learning SQL is attributable to the semantic transformation from NL to SQL. This proposal implies that understanding the planning-to-coding transformation is more difficult than understanding the formulation-to-planning transformation because the planning-to-coding transformation involves a semantic transformation from NL to SQL. Therefore, the following hypothesis is proposed:

Hypothesis: The semantic distance of the planning-to-coding transformation is greater than that of the formulation-to-planning transformation, which renders the planning-to-coding transformation more difficult to understand than the formulation-to-planning transformation.

## Research methodology

In this study, an empirical experiment was performed in a database course to investigate the influence of the semantic distance on SQL learning by comparing learners’ formulation-to-planning and planning-to-coding transformations.

### Experimental procedure

Most SQL learning studies evaluated learning outcomes through an SQL writing test which contains a set of data query questions and the ER model and relational schemas needed to answer those questions (Junkkari et al., 2016; Taipalus, 2020b). Learners’ understanding of SQL was measured by their answers to the questions. The present study used the same method to measure the students’ understanding of SQL. Considering the ability to access databases using SQL is a core competency of management information systems (MIS) majors, they were representative of the population that the present study intended to investigate. Thus, the experiment was performed in a database course in the Department of Information Science and Management Systems at National Taitung University in 2020. To avoid conclusion validity threats caused by random heterogeneity of the subjects (Wohlin et al., 2012), the following selection criteria were applied: (1) participants must be MIS majors and had taken the same courses, such as object-oriented programming and web programming; (2) they were willing to participate in this research. To ensure that the participants had similar levels of prior knowledge regarding SQL, a data query test was performed prior to the start of the course. The test revealed that the participants had no knowledge of databases. Finally, forty undergraduate students aged 19–21 years old were enrolled as participants in the experiment. The numbers of male and female participants were 27 (67.5%) and 13 (32.5%). Ethical approval was obtained from the National Cheng Kung University Human Research Ethics Committee (Reference Number NCKU HREC-E-108-274-2).

The course participants met for 3 h weekly for 18 weeks, including the midterm and final exams (2 weeks). The course first introduced basic database concepts (1 week), ER models (1.5 weeks), relational models (1.5 weeks), and the transformation of ER models into relational models (1 week). Then, the course introduced normalization of relations (1 week), relational algebra (1 week), structured query language (5 weeks), file organizations on disk and physical database design (1 week), query optimization (2 weeks), and transaction and concurrency (1 week). The SQL material covered data definition, control, and manipulation language. The data manipulation language material covered the syntaxes of select, update, insert, and delete. The instructor taught all types of syntaxes but focused heavily on the select syntax because when learning SQL, the select syntax is the most relevant and many of its concepts are used in other types of syntaxes. The following syntax concepts were covered: one-table simple query, function, order by, arithmetic operator, simple subquery, in, exists, inner join, outer join, self-join, correlated subquery, aggregate function, set operator, and group by with having.

At the end of the database course, the participants undertook a paper-based query-writing test in a classroom, where they were supervised by a research assistant throughout the test. The assistant supervised the test and was prepared to handle any problems that cropped up. The 3-h test comprised two segments. The participants were asked to perform a task for each segment, and the mental effort they expended to perform the task was then measured. Those who completed both segments were asked to submit their test results and leave the classroom.

### Measurement of understanding

Learners’ understanding of a course material is typically indicated by recall accuracy, response latency, problem-solving performance, and mental efficiency (Pinggera et al., 2015; Shin, 2020; Guo and Liao, 2022; Shanta and Wells, 2022). Mental efficiency and problem-solving performance are suitable measures of deep understanding, whereas Response latency and recall accuracy are suitable measures of superficial understanding (Van Gog and Paas, 2008). The author observed that most students can clearly understand individual SQL concepts but solving data queries by applying these concepts simultaneously was difficult for them. This implies that learners must deeply understand the complex semantics of SQL, instead of superficially understanding SQL syntax. Furthermore, the problem-solving performance measure has been commonly used in SQL learning research. Thus, problem-solving performance and mental efficiency were adopted in this study.

Problem-solving performance was measured in terms of query accuracy, which has been widely used in the research of SQL learning (Taipalus, 2020b). The query-writing test in this study covered two segments: (1) formulation-to-planning transformation: the participants had to write query logics of the data query questions by using their own words. (2) Planning-to-coding transformation: the participants had to transform the query logics into SQL statements. Query accuracy was scored using the widely used grading method developed by Siau et al. (2004). The data query questions were scored as essentially correct or incorrect. The essentially correct category included completely correct responses and responses with minor errors. Completely correct responses indicated correct data retrieval. Responses with minor errors contained errors, such as extra or omitted quotation marks, misspelled column names, and misspelled data values, that were minor and could be easily discovered. The answers that did not retrieve the correct data were rated as incorrect. A senior database professional and a MIS professor scored the answers of the participants. Inconsistencies between the raters were resolved through discussion and a review of the answers.

The mental efficiency of learners was measured using the approach developed by Paas and van Merriënboer (1993), the Eq. 1. The mental effort expended on each segment of the query-writing task was measured at the end of each segment by using the subjective rating scale (ranging from 1 = extremely low mental effort to 7 = extremely high mental effort). Studies have demonstrated that subjective measures of mental effort are highly correlated with objective measures (O’Donnell and Eggemeier, 1986) and are sensitive to minute differences in mental effort (Paas et al., 1994). Furthermore, people have no difficulty in complying with the request to assign a numerical value to the perceived mental effort imposed by tasks (Gopher and Braune, 1984). Therefore, the subjective rating scale of mental effort developed by Paas and van Merriënboer (1993) is considered valid and reliable. This approach standardizes mental effort and problem-solving performance scores into *z*-scores (*M* = 0, *SD* = 1) to calculate the mental efficiency score. Negative mental efficiency scores imply inefficient instruction conditions because the invested mental effort exceeds the performance output, while a positive score indicates the reverse.


(1) $$M\,e\,n\,t\,a\,l\,E\,f\,f\,i\,c\,i\,e\,n\,c\,y\,S\,c\,o\,r\,e=\frac{Z_{\mathrm{Problem}-\mathrm{solving}\,\mathrm{performance}}-Z_{\mathrm{Mental}\,\mathrm{effort}}}{\sqrt{2}}$$

### Test task

The query-writing task pertains to an e-bookstore system. Customers can view the products and place orders to buy the products. Appendices A, B present the ER diagram of the system and the data query questions, respectively. Figure 2 presents the relational schema. In the SQL learning literature, data query questions are typically divided into two or three levels according to difficulty. Jih et al. (1989) classified questions as simple and complex. Simple queries involved a single table, whereas complex queries involved a join across two tables. Siau et al. (2004) classified questions as simple, medium, and complex. They defined a simple query as a one-table query, a medium query as one with a join across two tables, and a complex query as one with more tables, more join operations or a nested operation. Considering the present study includes queries with joins and other operations such as nesting, the questions were categorized into three levels, namely easy, intermediate, and difficult. The easy questions were defined as one-table queries involving arithmetic operations, selection, projection, and/or operators, and functions. The intermediate questions were defined as two-table queries involving join, nesting, set functions, and group by. The difficult questions were defined as queries by using more nested operations, more join operations, more tables, and combinations of the syntaxes used in the easy and intermediate levels. Eighteen questions (six for each level) were developed on the basis of the above criteria. To ensure the validity of the task, two database instructors and two senior database experts were hired to review the test materials, and some revisions were made to the materials. Thereafter, the task was tested in a pilot test to rectify any ambiguity and unclarity. Nine undergraduates who majored in MIS were recruited for the pilot test. They had completed the database course before the test. The results indicated that the task materials were reasonable and the expression is accurate.

### Validity threats

This section discusses how the conclusion validity, internal validity, construct validity, and external validity of this study were addressed.

Conclusion validity refers to the relationship between the treatment and experimental results (Wohlin et al., 2012). The present study addressed three possible threats to conclusion validity, namely, the reliability of the instruments, the size of the sample, and the random heterogeneity of the participants. The basic principle of instrument reliability is that when you measure a phenomenon twice, the result should be the same. The reliability of an instrument depends on many factors. For example, objective measures that can repeat the same results are more reliable than subjective measures (Wohlin et al., 2012). In the present study, query accuracy was scored according to whether the answer retrieved the correct data; that is, query accuracy was objectively determined, thus ensuing reliability. With regard to the threat from sample size, the sample was enough for conclusion validity to be achieved in a paired samples *t*-test. Finally, the threat to the random heterogeneity of the participants was avoided. The reasons have been explained in section “Experimental procedure.”

Internal validity refers to the reliability of the study results obtained in a given environment (Wohlin et al., 2012). This study addressed three possible internal validity threats, namely, mortality-related, history-related, and testing-related threats. Mortality-related threats to internal validity are present when participants leave a study. In the present study, no participant left the experiment. Therefore, this threat was absent. History-related threats are present when the treatment received by participants differs between time points. In the present study, history-related threats were avoided because only one treatment was applied. Finally, testing-related threats are present when a test is repeated in an experiment because participants are learning the testing procedure. In the present study, they were avoided because only one test was performed.

Construct validity pertains to how accurately a measure measures the concept it is intended to measure (Wohlin et al., 2012). To ensure that the measures accurately reflect SQL learning outcomes, the present study used mental efficiency and problem-solving performance (instead of recall accuracy and response latency) to measure the participants’ understanding of SQL; these measures were used because SQL learning involves complex semantic transformations, and problem-solving performance and mental efficiency are suitable measures of deep understanding (Van Gog and Paas, 2008). The present study addressed two other construct validity threats, namely, the interaction of multiple treatments and the confounding of constructs with levels of constructs. The threat posed by interactions among multiple treatments concerns whether the effect of an experiment can be ascribed to interactions with the treatments of other experiments. Because the participants in the present study did not participate in other studies, this threat could be ruled out. The threat of confounding constructs with levels of constructs was addressed during the design of the data query questions. Specifically, the questions were classified as difficult, intermediate, and easy on the basis of their level of difficulty.

External validity pertains to the generalizability of a study’s findings outside the context of the study (Wohlin et al., 2012). The present study addressed two threats to external validity, namely, the setting-related and participant-related threats. Setting-related threats are present when the experiment is conducted in an unsuitable environment. The present research was conducted in the context of a university-based database course, which was supervised by an instructor who was a database professional. Thus, its findings are at least valid for learning SQL in universities. Participant-related threats are present when a participant population is not representative of the population. The participants were representative of the population that the present study intended to investigate. The reasons have been explained in section “Experimental procedure.”

## Results

The collected data were coded and entered into the SPSS data sheet for analysis. The paired samples *t*-test was used to determine whether the change in means between the two paired observations (the formulation-to-planning transformation task and the planning-to-coding transformation task) is statistically significant using SPSS (selecting “Analyze – compare means – paired samples *t* test”). The main results are presented in Tables 2, 3, and discussed below.

**TABLE 2:** Mental effort and problem-solving performance scores–mean (standard deviation) and paired samples *t*-test (*p*).

Dependent variable	Formulation-to-planning	Planning-to-coding	Paired samples *T*-test (*p*)
Problem-solving performance	14.575 (2.263)	10.625 (2.487)	26.795*** (0.000)
Mental effort	4.150 (1.272)	5.875 (0.965)	−19.689*** (0.000)

***p < 0.001.

**TABLE 3:** Standardized problem-solving performance, standardized mental effort, and mental efficiency scores–mean (standard deviation) and paired samples *t*-test (*p*).

Formulation-to-planning			Planning-to-coding			
Performance	Mental effort	Mental efficiency	Performance	Mental effort	Mental efficiency	Paired samples *T*-test (*p*)
0.63 (0.732)	−0.60 (0.896)	0.882 (0.253)	−0.63 (0.805)	0.60 (0.68)	−0.882 (0.285)	26.082*** (0.000)

***p < 0.001.

For the problem-solving performance measure, as indicated in Table 2, a statistically significant difference was observed between the formulation-to-planning transformation task (*M* = 14.575, *SD* = 2.263) and the planning-to-coding transformation task (*M* = 10.625, *SD* = 2.487) with *t*(40) = 26.795 and *p* < 0.001, implying that the participants exhibited greater problem-solving performance in the formulation-to-planning transformation task than in the planning-to-coding transformation task. For the mental effort measure, a statistically significant difference was also observed between the formulation-to-planning transformation task (*M* = 4.150, *SD* = 1.272) and the planning-to-coding transformation task (*M* = 5.875, *SD* = 0.965) with *t*(40) = −19.689 and *p* < 0.001, implying that the participants exerted greater mental effort in the planning-to-coding transformation task than in the formulation-to-planning transformation task.

The mental efficiency scores of the participants were calculated from the standardized *z*-scores of their mental effort and problem-solving performance scores. As indicated in Table 3, a statistically significant difference was observed between the formulation-to-planning transformation task (*M* = 0.882, *SD* = 0.253) and the planning-to-coding transformation task (*M* = −0.882, *SD* = 0.285) with *t*(40) = 26.082 and *p* < 0.001, implying that the participants exerted less mental effort and exhibited superior problem-solving performance in the formulation-to-planning transformation task than in the planning-to-coding transformation task, which indicated that understanding the planning-to-coding transformation is more difficult than understanding the formulation-to-planning transformation.

## Discussion

This section first analyzes the influence of semantic distance on SQL learning based on the data in Tables 2, 3, and then further discusses the implications of the research results.

### Influence of semantic distance on structured query language learning

The learners demonstrated a higher level of problem-solving performance (as shown in Table 2) and mental efficiency (as shown in Table 3) in the formulation-to-planning transformation task than in the planning-to-coding transformation task. The results of the data analysis support the hypothesis that it is more difficult to understand the planning-to-coding transformation because the semantic distance of the planning-to-coding transformation is greater than that of the formulation-to-planning transformation. Only NL was used in the formulation stage and planning stage to describe data queries. By contrast, different languages, namely NL and SQL, were used in the planning stage and coding stage, respectively. This implies that the learners were able to use NL to write a query logic for a data query (i.e., formulation-to-planning transformation), but it was difficult for them to translate the logic into an SQL statement (i.e., planning-to-coding transformation) because the planning-to-coding transformation involved a semantic transformation from NL to SQL, which are two essentially different languages. This difference was reflected in two characteristics: expressive ease versus low expressive ease and procedural versus declarative nature.

(A) Expressive ease versus low expressive ease: Expressive ease refers to the syntactic flexibility permitted in the formulation of data queries (Taipalus and Seppänen, 2020). Studies have indicated that query languages with high levels of expressive ease can free learners from the constraints of syntactic details (Miedema and Fletcher, 2021). In this condition, learners can concentrate on reasoning out data queries and formulating appropriate solutions to seek their answers instead of ensuring syntactic correctness. NL has a higher expressive ease than SQL because SQL has a restricted syntax with a limited set of keywords (Elmasri and Navathe, 2016).

(B) Procedural versus declarative nature: NL can be used as a procedural language, whereas SQL is pure declarative. Procedural languages, such as Java, can break a problem into simpler sub-problems that can be solved in several steps, whereas declarative languages can only indicate the requirements of a problem but cannot specify how these requirements are to be achieved (Sadiq et al., 2004). For example, in the planning stage, learners can use NL to describe the steps of a query logic, for instance, how to obtain the initial datasets from the relations, and process them through operations to obtain the final resultant dataset. By contrast, in the coding stage, learners use SQL to implement the query logic. However, they cannot specify the execution steps of the SQL statements, and worse, these steps are not visible. They must visualize and conceptualize the intermediate datasets of SQL statements and the entire process in terms of SQL syntax in their working memory (Lavbič et al., 2017). Thus, NL and SQL are essentially different in terms of the two aforementioned characteristics. In the light of the semantic network theory, the differences between NL and SQL imply that the establishment and retrieval of the semantic relations from semantic memory are more difficult in case of the planning-to-coding transformation than in case of the formulation-to-planning transformation (de Barros Pereira et al., 2022).

The establishment of semantic relations: to meaningfully learn a semantic relation of the planning-to-coding transformation, learners must assimilate the learned SQL syntax into their existing knowledge structures (Chen et al., 2013). Meaningful learning is when learners can create nodes for new information and related them to the nodes associated with already learned knowledge in their existing knowledge structure. However, learners may encounter difficulties in establishing semantic relations between the planning stage and the coding stage because the types of knowledge used in the two stages are markedly different. The planning and coding stages use NL and SQL, respectively. NL and SQL belong to distinct knowledge categories. According to the semantic network theory, they share fewer properties in an underlying semantic structure and are semantically less mutually relevant (Gonzalez, 2019). Thus, it is relatively difficult to establish semantic relations between the two stages. In this context, learners are forced to memorize each newly learned SQL concept by rote as a separate item to be added into semantic memory, which may impair meaningful learning (Mayer, 1981).

The retrieval of semantic relations: when learners retrieve semantic relations that they have learned from their knowledge structure to resolve a data query, they prime the nodes in the semantic memory to retrieve the relations. Semantic network theory indicates that the accuracy of recalling semantic relations is proportional to the strength of priming (Ashcraft, 2002). The strength of priming decays exponentially with the distance over which it spreads, thereby reducing the ability to recall semantic relations (Ortony et al., 2022). Thus, the accuracy with which a semantic relation is recalled is inversely proportional to the semantic distance of the relation (Gonzalez, 2019). In the context of SQL learning, the types of knowledge required in the planning and coding stages belong to distinct knowledge categories. According to the semantic network theory, the links that connect the nodes of the concepts representing the types of knowledge associated with the two stages in the semantic network are distant and indirect (de Barros Pereira et al., 2022). Thus, recalling the semantic relations of the planning-to-coding transformation is more difficult than recalling the semantic relations of the formulation-to-planning transformation. A higher semantic recall implies a superior understanding of the learning material (Gemino and Wand, 2004). Accordingly, it is more difficult to understand the semantic relations of the planning-to-coding transformation than those of the formulation-to-planning transformation.

In summary, the study results imply that the semantic distance of the planning-to-coding transformation is greater than that of the formulation-to-planning transformation. The large semantic distance of the planning-to-coding transformation leads to the difficulty in establishing and retrieving the semantic relations from the semantic memory. This difficulty increases learners’ cognitive load, thereby jeopardizing their learning outcomes (Thees et al., 2020; Ayres et al., 2021). This is supported by the experimental result that the participants invested more mental effort, but exhibited poorer problem-solving performance in the planning-to-coding transformation task than in the formulation-to-planning transformation task. These results are consistent with previous empirical evidence on SQL learning that the mental effort required to formulate data queries increases as the semantic distance increases (Borthick et al., 2001). Learning semantic transformations with shorter semantic distances requires less mental effort and leads to better learning outcomes (Rho and March, 1997). Thus, SQL teaching methods should strengthen the semantic mapping of the planning-to-coding transformation in learning materials and shorten the semantic distance to facilitate learners’ understanding of SQL.

### Recommendations

The findings of this study suggest that pedagogical methods should (1) focus on the semantic transformation from the planning stage to the coding stage and (2) shorten the semantic distance involved in learning SQL. Below are suggestions for teaching methods that can reduce the influence of semantic distance on SQL learning.

To follow the first principle, the following tools can be used: Animated SQL tutoring systems (e.g., eSQL, SAVI, and DBQA), SQL query generators (e.g., SiS, SQL Developer, and SQL visualizer), and concept-map-based SQL instruction. Animated SQL tutoring systems emphasize the semantic transformation from the planning to coding stage by illustrating initial datasets, the evolution of these datasets into intermediate datasets, and the final transformation of these datasets into the resultant dataset. SQL query generators divide the SQL query building process into several steps. The changes made to each step during the process lead to the generation of the corresponding output dataset and SQL statement. Both types of teaching methods illustrate the transformation from the planning to coding stage, but in different ways. Illustrations of the process of SQL execution can underscore the key concepts underlying SQL queries and focus the attention of learners on semantic transformation, thereby enhancing their understanding of SQL (Wu et al., 2012). This finding is consistent with those of other studies; that is, graphic organizers are effective techniques for motivating learners (Stull and Mayer, 2007). Graphical representations allow learners to learn with a low extraneous cognitive load because information is presented in a comprehensive and holistic manner (Sweller et al., 2011).

The concept-map-based SQL instruction strengthens the semantic transformation from the planning stage to the coding stage by representing the transformation with concept maps. When establishing a concept map for learning a planning-to-coding transformation, learners are required to reflect on the semantic mapping between the two stages, leading them to relate the new SQL knowledge to what they already know through reviewing, adding on to, or modifying their current knowledge of the semantic mapping (Wu et al., 2012). Studies have indicated that constructing concept maps can promote meaningful learning (Erdogan, 2009; Roessger et al., 2018). Teaching methods that emphasize the relationship between learners’ existing knowledge and the newly learned knowledge can promote meaningful learning (Novak and Cañas, 2006). When studying instructors’ concept maps, learners are guided to implement the SQL query by following the cognitive process used by the instructor, which helps learners to easily understand the semantic transformation process. Furthermore, by comparing the similarities between learners’ concept map and instructors’ concept map, learners can identify their errors and thus improve their understanding (Chen et al., 2013).

To follow the second principle, the following tools can be used: SQL query generators, divide-and-conquer method (Qian, 2018), and concept-map-based SQL instruction. SQL query generators enable learners to learn the semantic transformation of data requests into SQL statements in a step-by-step format, whereas conventional teaching methods teach learners how to transform data requests directly into SQL statements. SQL query generators reduce the semantic distance of each step, thereby reducing the cognitive load required for learners to learn semantic transformation and enhancing their comprehension (Ortony et al., 2022). Similarly, the divide-and-conquer method satisfies the second principle. This method decomposes a data request into subrequests and then combines them to reconstruct the original SQL query. The semantic distance of subrequests is shorter than that of the original SQL query. The concept-map-based SQL instruction divides the semantic transformation into two segments, namely the formulation-to-planning transformation and planning-to-coding transformation. Learners learn the formulation-to-planning transformation and subsequently learn the planning-to-coding transformation. Therefore, learners who receive the concept-map-based SQL instruction have a short semantic distance in each segment. From the viewpoint of semantic network theory, the concept-map-based SQL instruction may promote the understanding of SQL because the semantic distance of learning instructions and the understanding is positively related (Ashcraft, 2002). The current study provided preliminary principles from the viewpoint of semantic network theory to explain how an SQL teaching method assists in understanding SQL.

## Conclusion

Students of database courses encounter barriers when learning SQL. The results of the present study indicated that the difficulties associated with SQL learning were mainly attributable to the greater semantic distance of the planning-to-coding transformation than that of the formulation-to-planning transformation. The establishment and retrieval of the semantic relations of the planning-to-coding transformation in semantic memory are difficult. The study results lay a theoretical foundation for explaining SQL learning difficulties from the perspective of semantic distance by using semantic network theory. Accordingly, the preliminary principles for evaluating the effectiveness of SQL teaching methods in assisting SQL learning were established in this study: (1) strengthening the semantic mapping between the planning stage and the coding stage; (2) shortening the semantic distance involved in learning SQL. These principles will help educators realize the SQL learning difficulties caused by semantic transformation, focus on areas where learners must strengthen their understanding, and motivate researchers to develop more effective teaching methods from the viewpoint of semantic network theory.

Although this study provides insights into the effects of semantic distance on SQL learning and may help promote the further development of SQL pedagogies, only one test task was investigated. Therefore, future studies should include more tasks to obtain further information. Furthermore, this research is but a first step toward a comprehensive understanding of the cognitive processes of SQL learners. More in-depth follow-up studies are required. Multiple instructors and diverse experimental designs should be used in future research.

## Data availability statement

The raw data supporting the conclusions of this article will be made available by the authors, without undue reservation.

## Ethics statement

The studies involving human participants were reviewed and approved by the National Cheng Kung University Human Research Ethics Committee. The patients/participants provided their written informed consent to participate in this study.

## Author contributions

The author confirms being the sole contributor of this work and has approved it for publication.

## Figures and Tables

**FIGURE A1:**
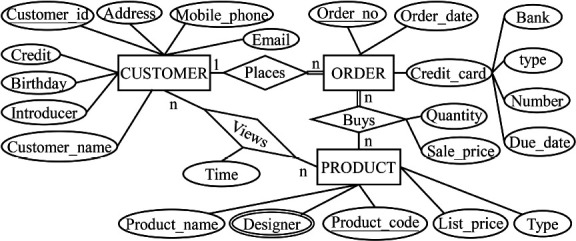
ER diagram of the e-bookstore system.

## Appendix

### Appendix B: Data query questions

**Easy**

1. Retrieve the names and mobile phone numbers of all the customers.
2. Retrieve the codes and names of the products with a list price of 400–900.
3. Retrieve the identities of the customers who were not introduced.
4. Retrieve the names of the customers who have the same birthday as the customer Michael.
5. Retrieve the names of the customers who live in New York.
6. Retrieve the code of the product that has the lowest sale price.

**Intermediate**

1. Retrieve the codes of the CD products created by John.
2. Retrieve the names of the customers who have placed orders.
3. Retrieve the names of the customers who placed order a82222.
4. Retrieve the names and email addresses of the customers who have placed more than one order.
5. Retrieve the names of the customers who viewed product d40111.
6. Retrieve the identities and names of all the customers and the name of their introducer.

**Difficult**

1. Retrieve the names of the customers who have bought product c41888.
2. Retrieve the numbers of the customers who have purchased product d50111.
3. Retrieve the names of the customers who have bought the products that were bought by customer d0811457.
4. Retrieve the identities of the customers who have exceeded their credit limits.
5. Retrieve the codes of the products that have been bought more than 10 items.
6. Retrieve the orders whose order quantity is greater than twice the average order quantity, showing the order numbers, customers’ identities, and customers’ names.
